# The Basic Helix-Loop-Helix Transcription Factor Family in the Honey Bee, *Apis mellifera*

**DOI:** 10.1673/031.008.4001

**Published:** 2008-05-20

**Authors:** Yong Wang, Keping Chen, Qin Yao, Wenbing Wang, Zhi Zhu

**Affiliations:** ^1^ Department of Biotechnology, Faculty of Food and Biological Engineering, Jiangsu University, 301 Xuefu Road, Zhenjiang 212013, P. R. China; ^2^ Institute of Life Sciences, Jiangsu University, 301 Xuefu Road, Zhenjiang 212013, P. R. China

**Keywords:** phytogeny, blast search

## Abstract

The basic helix-loop-helix (bHLH) transcription factors play important roles in a wide range of developmental processes in higher organisms. bHLH family members have been identified in a dozen of organisms including fruit fly, mouse and human. In this study, we identified 51 bHLH sequences *in silico* in the honey bee, *Apis mellifera* L. (Hymenoptera: Apidae), genome. Phylogenetic analyses revealed that they belong to 38 bHLH families with 21, 11, 9, 1, 8 and 1 members in high-order groups A, B, C, D, E and F, respectively. Using phylogenetic analyses, all of the 51 bHLH sequences were assigned to their corresponding families. Genes that encode ASCb, NeuroD, Oligo, Delilah, MyoRb, Figa and Mad were not found in the honey bee genome. The present study provides useful background information for future studies using the honey bee as a model system for insect development.

## Introduction

The basic helix-loop-helix (bHLH) family of transcription factors plays important roles in a wide range of developmental processes including neurogenesis, myogenesis, hematopoiesis, sex determination and gut development (Massari and Murre 2000). Since the first characterization of the mouse bHLH transcription factors E12 and E47 (Murre et al. 1989), hundreds of bHLH proteins have been identified so far. In 1999, Atchley et al developed a predictive motif for the bHLH domains based on amino acid frequencies at all positions of 242 bHLH proteins (Atchley et al. 1999). 19 conserved sites were found within the bHLH domain. Atchley et al. (1999) showed that a sequence with less than 8 mismatches to the predictive motif was very possibly a bHLH protein. Later, researchers found that a sequence even with 9 mis-matches could also be a potential bHLH protein (Toledo-Ortiz et al. 2003). In recent years, more bHLH genes have been identified in organisms whose genome sequences were available. These include 8 bHLH genes in yeast, 16 in *Amphimedon queenslandica*, 33 in *Hydra magnipapillata*, 39 in *Caenorhabditis elegans*, 39 in *Callus gallus*, 39 in *Brachydanio rerio*, 46 in *Ciona intestinalis*, 47 in *Xenopus laevis*, 50 in *Strongylocentrotus purpuratus*, 57 in *Daphia pulex*, 59 in *Drosophila melanogaster*, 63 in *Lottia gigantea*, 64 in *Capitella* sp 1, 68 in *Nematodtella vectensis*, 78 in *Branchiostoma floridae*, 87 in pufferfish, 102 in mouse, 118 in human, 147 in *Arabidopsis* and 167 in rice (Ledent et al. 2002; Li et al. 2006; Satou et al. 2003; Simionato et al. 2007; Toledo-Ortiz et al. 2003). Based on phylogenetic analyses of over 400 bHLH proteins, Ledent et al defined 45 orthologous families and 6 higher-order groups for all the identified bHLH genes, where the 44 families were named according to their name of the first discovered or best-known member of the family, and the higher-order groups were named A to F based on the different properties of these groups (Atchley and Fitch 1997; Ledent et al. 2002; Ledent and Vervoort 2001; Simionato et al. 2007). Groups A and B bHLH proteins bind to core DNA sequences typical of E boxes (CANNTG) which is CACCTG or CAGCTG for group A and CACGTG or CATGTTG for group B. Group C comprises the family of bHLH proteins known as bHLH-PAS because a PAS domain follows the bHLH motif. The core sequences to which they bind are ACGTG or GCGTG, while recent studies have demonstrated that the *Drosophila* Dysfusion/ Tango bHLH-PAS heterodimer has a binding preference as TCGTG > GCGTG > ACGTG > CCGTG (Jiang and Crews 2007). Group D proteins lack a basic domain. They are not able to bind DNA. They function as antagonists of group A bHLH proteins. Group E proteins are mainly related to the Drosophila Hairy and E(spl) bHLH proteins. These proteins bind preferentially to sequences typical of N boxes (CACGCG or CACGAG). They also contain two additional domains named ‘Orange’ and WRPW peptide in the carboxyl-terminal part. Group F proteins have the COE domain which is characterized by the presence of an additional domain involved both in dimerization and in DNA binding (Ledent and Vervoort 2001).

The honey bee, *Apis mellifera* L. (Hymenoptera: Apidae), is a key model for social behavior. Many studies have been conducted to elucidate the developmental processes that result in its particular social organization. However, not many bHLH transcription factors have been characterized. So far, seven honey bee bHLH sequences have been reported. They are AmCYC and AmCLK for which cDNA sequences were cloned (Rubin et al. 2006), two Achaete-Scute genes and three Enhancer of split genes that were identified in the honey bee genome (Schlatter and Maier 2005). The latest version of honey bee genome sequence has been available in the GenBank since October 2007. In this study, we used both the representative sequences of the 45 bHLH families (Ledent and Vervoort 2001) and the known 59 *Drosophila melanogaster* bHLH (DmbHLH) sequences (Ledent et al. 2002; Simionato et al. 2007) to conduct tblastn searches against database of the *Apis mellifera* genome sequences. After examining the amino acid residues at the 19 conserved sites, we found that 51 *Apis mellifera* bHLH (AmbHLH) sequences satisfied the screening criterion. Phylogenetic analyses with the 45 representative bHLH domains and with the 59 DmbHLH sequences defined the families to which the 51 AmbHLH sequences belong.

## Materials and Methods

### tblastn searches

The sets of 45 representative bHLH domains and 59 DmbHLH motifs were from the additional files of (Ledent and Vervoort 2001) and (Simionato et al. 2007), respectively. Each sequence of both sets was used to perform tblastn searches against the database of *Apis mellifera* genome draft sequences (http://www.ncbi.nlm.nih.gov/genome/seq/BlastGen/BlastGen.cgi?taxid=7460). Tblastn searches compare a protein query sequence against a nucleotide sequence database dynamically translated in all six reading frames of both strands. Stringency was set as *E* < 10 in order to obtain all bHLH related sequences for later examination.

### Manual improvement to the obtained sequences

The obtained subject sequences from the tblastn searches were examined manually to keep only one sequence for those that have the same scaffold number, reading frame and coding regions. Manual improvement was also done to the sequences lacking a few amino acids on their two ends. This was realized by retrieving the whole subject sequence from GenBank and translating it with EditSeq program (version 5.01) of the DNAStar package to obtain the absent amino acid residues. To those subject sequences that had coding regions separated by dozens to thousands of nucleotides, the SpliceView application (http://www.itb.cnr.it/sun/webgene/) was used to analyze if the sequences had introns.

**Figure 1.**
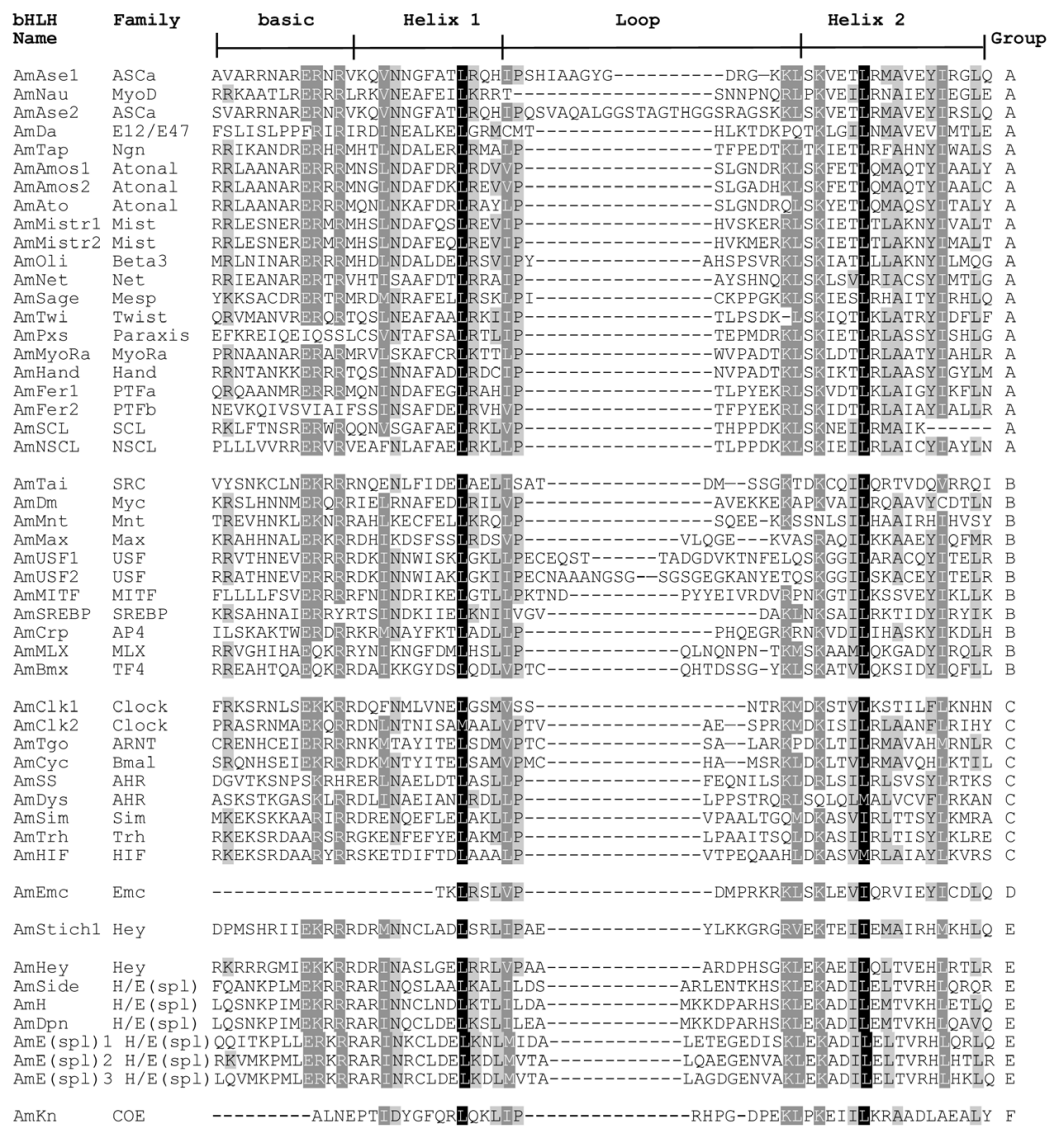
Alignment of 51 AmbHLH members. Designation of basic, helix 1, loop and helix 2 follows Ferre-D'Amare et al. (Ferre-D'Amare 1993). The family names and high-order groups have been organized according to Table 1 in Ledent et al (Ledent et al. 2002). AmbHLHs were named in accordance with fruit fly nomenclature.

### Sequence alignment

All sequences that had undergone the above improvement were aligned using ClustalW online (http://www.ebi.ac.uk/clustalw/) with default settings. The aligned sequences were transferred into a Microsoft Excel worksheet for examining the amino acid residues at the 19 conserved sites at specific sites. Sequences with less than 9 variations were regarded as potential AmbHLHs and were aligned again using ClustalW. The aligned AmbHLHs were shaded in GeneDoc Multiple Sequence Alignment Editor and Shading Utility (Version 2.6.02) (Nicholas et al. 1997) and copied to a Word RTF file for further annotation.

### Phylogenetic analyses

Phylogenetic analyses were conducted using PAUP 4.0 Beta 10 (Swoffbrd 1998) based on a stepmatrix constructed from Dayhoff PAM 250 distance matrix by R. K. Kuzoff (http://paup.csit.fsu.edu/nfiles.html). The obtained AmbHLH sequences were used to construct neighbor-joining distance trees with the 45 representative bHLH domains and with the 59 DmbHLH motifs, respectively. Sequences were first aligned in ClustalW and then copied into PAUP window to prepare nexus files. Neighbor joining trees were bootstrapped with 1,000 replicates to provide information about their statistical reliability. Maximum parsimony trees were constructed using PAUP 4.0 Beta 10 by executing command “bootstrap nreps = 100 search = heuristic/addseq = random”. Other parameters were set to default values. Maximum likelihood trees were constructed using TreePuzzle 5.2 (Schmidt et al. 2002). The number of puzzling steps was set to 25,000. Model of substitution was set to Jones-Taylor-Thornton Jones et al. 1992). Other parameters were default values. The trees were displayed using the Tree View program (version 1.6.6) (http://taxonomy.zoology.gla.ac.uk/rod/treeview.html), saved as Phylip format, edited using MEGA3.1 (Kumar et al. 2004), copied to clipboard, and then annotated in Microsoft PowerPoint.

**Table 1.**
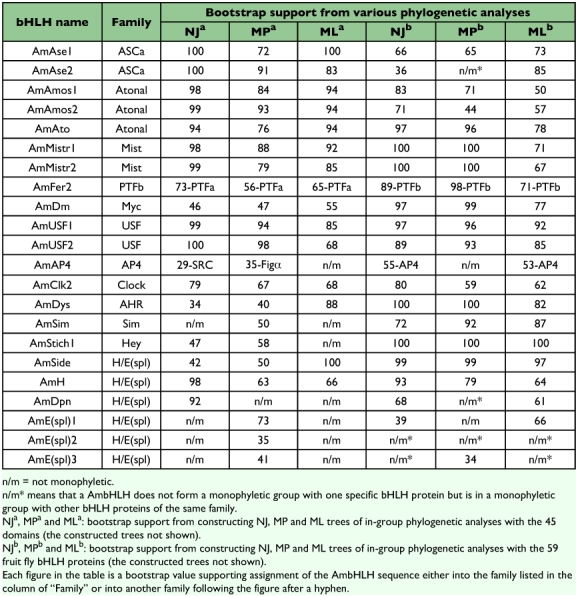
Assignment of AmbHLH members into corresponding families.

#### EST searches

In order to find existing expressed sequence tags (ESTs) matching the obtained AmbHLH sequences, tblastn searches were performed against honey bee EST database on NCBI tblastn website using each AmbHLH as the query sequence. The stringency was set as *E* < 0.0001. A 90% or higher identity was considered to be an EST corresponding to the specific AmbHLH sequence.

**Table 2.**
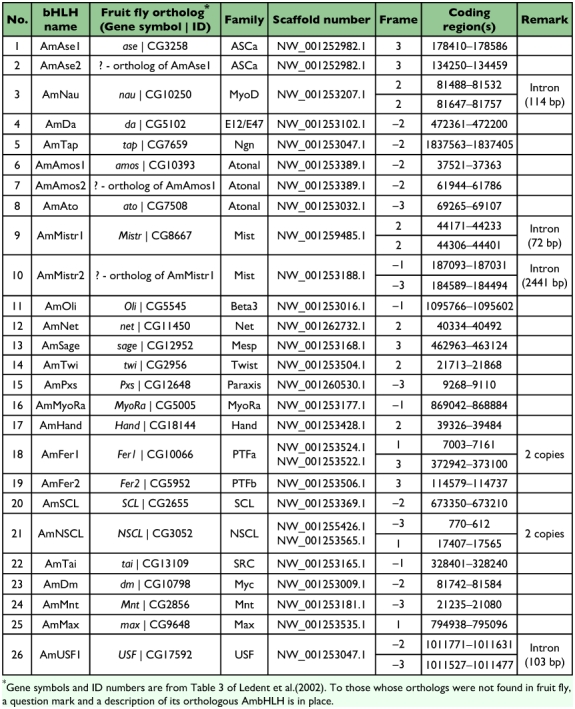
Coding regions of 51 AmbHLH domains.

**continued:**
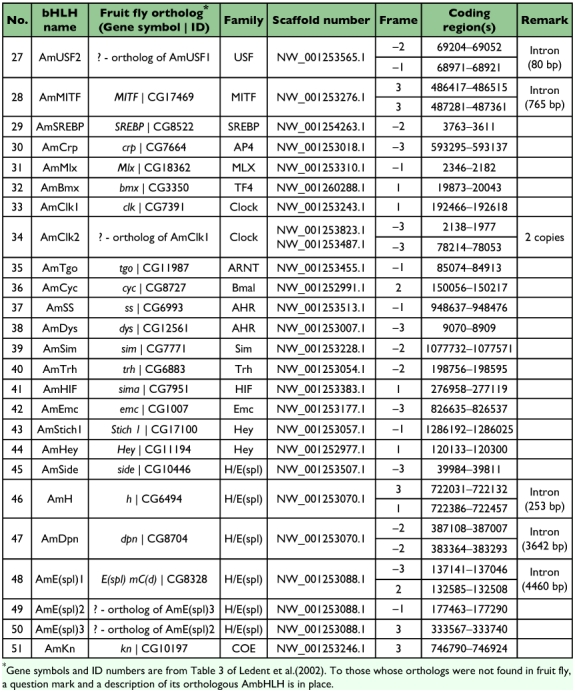

### Results and Discussion

#### Identification of AmbHLH sequences in the *A. mellifera* genome database

**Figure 2.**
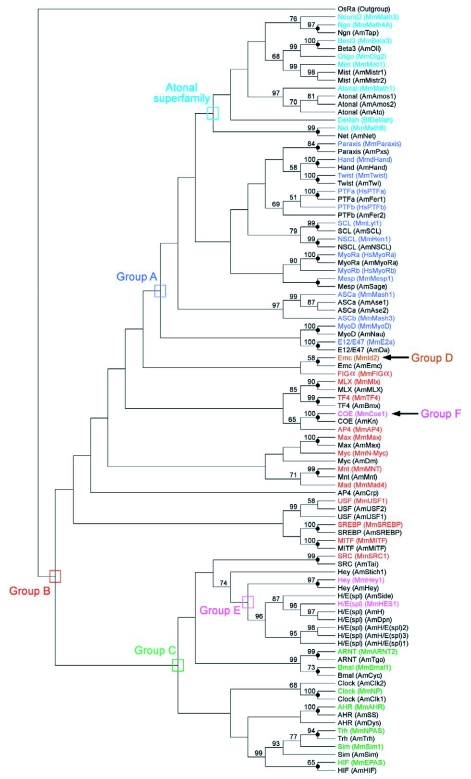
Phylogenetic relationship of 51 AmbHLH members with 45 bHLH domains. A neighborjoining (NJ) tree is shown. For simplicity, branch lengths of the tree are not proportional to distances between sequences, and bootstrap values less than 50 are not shown. The higher-order group labels are in accordance with (Ledent et al. 2002).

**Figure 3.**
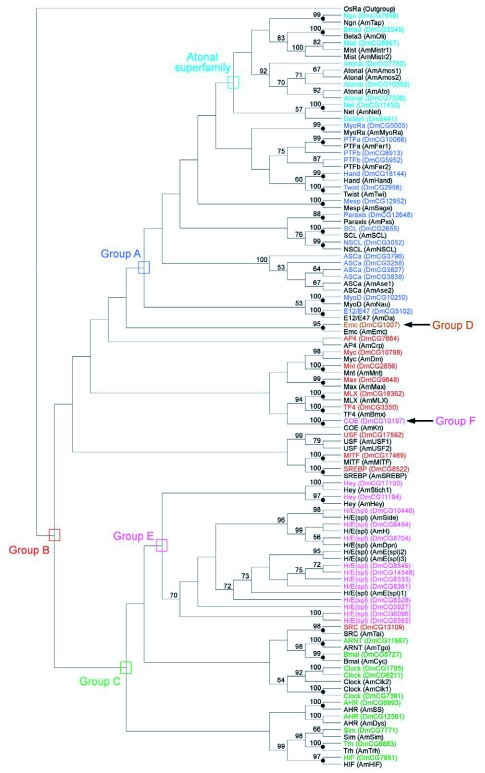
Phylogenetic relationship of 51 AmbHLH members with 59 *Drosophila* bHLHs. A neighbor-joining (NJ) tree is shown. For simplicity, branch lengths of the tree are not proportional to distances between sequences, and bootstrap values less than 50 are not shown. The higher-order group labels are in accordance with (Ledent et al. 2002).

Tblastn searches with the 45 bHLH domains and 59 DmbHLH motifs followed by manual improvement and examination led to the identification of 50 and 1 potential AmbHLH sequences, respectively. The alignment of all 51 AmbHLH domains is shown in Figure 1. Since there had been sufficient bootstrap support in the following phylogenetic analyses, the AmbHLHs were named according to their orthologs in *D. melanogaster*. Data supporting this nomenclature are provided in Figures 2, 3 and Table 1. The *D. melanogaster* orthologs are listed in Table 2 for reference. All of the phylogenetic analyses revealed that the 51 AmbHLHs belong to 38 families with 21, 11, 9, 1, 8 and 1 members in groups A, B, C, D, E and F, respectively (Figure 1).

#### Identification of orthologous families

Identification of orthologous genes has been uncertain since there is no absolute criterion that can be used to decide if two genes are orthologous (Ledent and Vervoort 2001). Based on the criterion used by Ledent et al (Ledent et al. 2002; Ledent and Vervoort 2001), a more stringent criterion was used: a single AmbHLH must form a monophyletic group with another bHLH of a known family in phylogenetic trees constructed with different methods, and all the bootstrap values must exceed 50.

The obtained 51 AmbHLH sequences had been used to construct neighbor joining trees with 45 bHLH domains (Figure 2) and with 59 DmbHLH motifs (Figure 3), respectively. In both trees, OsRa (the rice bHLH sequence of R family) sequence was used as outgroup. In both Figures 2 and 3, it can be seen that 29 out of 51 AmbHLH sequences had formed monophylogenetic groups with 29 other bHLH sequences, respectively. Their bootstrap values ranged from 56 to 100. These 29 AmbHLHs are AmTap, AmOli, AmNet, AmPxs, AmHand, AmTwi, AmMyoRa, AmSCL, AmNSCL, AmSage, AmFer1 and AmNau of group A, AmSREBP, AmMLX, AmBmx, AmDm, AmMax, AmMITF, AmMnt and AmTai of group B, AmClk1, AmTgo, AmCyc, AmSS, AmTrh and AmHIF of group C, AmEmc of group D, AmHey of group E, and AmKn of group F, all of which nodes are indicated with black dots in Figures 2 and 3).

In order to define families for the rest 22 AmbHLHs, each of them was used to construct neighbor joining, maximum parsimony and maximum likelihood phylogenetic trees within the members of a particular higher-order group. The results are summarized in Table 1 (the constructed trees are not shown). Table 1 shows that, by constructing phylogenetic trees using a single AmbHLH sequence with other bHLH proteins belonging to the same higher-order group (termed “in-group” analysis), all of the 22 AmbHLH sequences can be assigned to specific bHLH families. It should be noted that not all of the bootstrap values are over 50 for each assignment. However, with the bootstrap values from the construction of six in-group phylogenetic trees, there was sufficient support to make the assignments shown in Table 1.

**Table 3.**
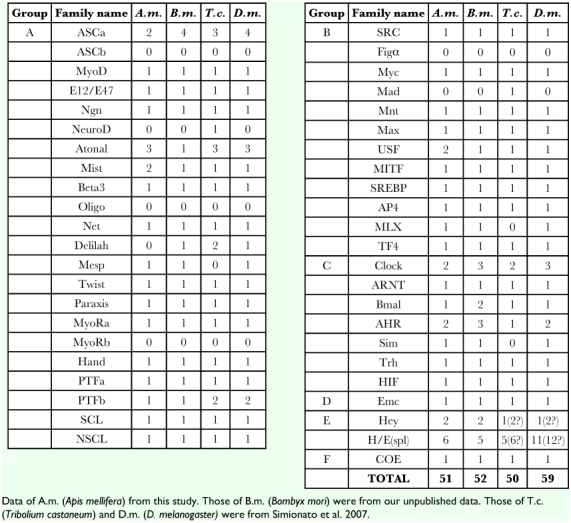
The insect bHLH members

The above phylogenetic analyses also enabled us to identify *D. melanogaster* orthologs for 44 AmbHLHs (Table 2). The remaining 7 AmbHLHs, namely AmAse2, AmAmos2, AmMistr2, AmUSF2, AmClk2, AmE(spl)2 and AmE(spl)3, did not form monophyletic groups with any *D. melanogaster* bHLHs. Instead, they formed monophyletic groups with other AmbHLHs as indicated with a question mark followed by the description of its orthologous AmbHLH. This result strongly suggests that these 7 AmbHLHs arose after *A. mellifera* diverged from the other insects.

**Figure 4.**
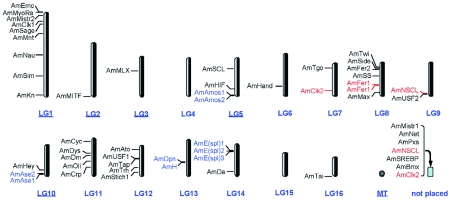
Localization of the AmbHLH coding regions. The AmbHLH names in red are those having two copies in the genome. The AmbHLH names in blue are those of the same family cluster together.

#### Coding regions and the localization of AmbHLH motifs

The coding regions for all the identified AmbHLH motifs are listed in Table 2. The data indicate that 9 AmbHLHs have introns in their bHLH motifs, among which AmNau, AmMistr1 and AmMistr2 have introns in helix 1 region, AmMITF, AmH, AmDpn and AmE(spl) 1 have introns in the loop region, and AmUSF1 and AmUSF2 have introns in helix 2 region. The length of the introns ranged from 72 to 4460 base pairs. It was also found that three AmbHLHs had 2 copies in the genome. They are AmFer1, AmNSCL and AmClk2.

Searches with the scaffold numbers listed in Table 3 in the honey bee map view (http://www.ncbi.nlm.nih.gov/ sites/entrez?db=genomeprj&cmd=Retrieve&dopt=Overview&list_uids=9555) located the positions of sequences coding for all of the AmbHLHs (Figure 4),which shows that the distribution of AmbHLH genes is fairly uneven. Chromosomes 1 and 8 have 9 and 7 AmbHLH genes, respectively. Chromosomes 11 and 12 have 5 AmbHLH genes each. Those on chromosomes 2, 3, 5, 6, 7, 9, 10, 13, 14 and 16 vary between 1 and 4. Chromosomes 4 and 15 and mitochondrial DNA do not code for any bHLH proteins. It should be noted that 7 AmbHLHs have not been placed in the map. It can also be seen in Figure 4 that two or three members of the same bHLH family often cluster together. For example, AmAmos1 and AmAmos2, AmAse1 and AmAse2, AmH and AmDpn, and AmE(spl)1, 2 and 3 all locate on the same scaffold, respectively (indicated in blue). This suggests a possible origination for the other member by gene duplication.

#### The AmbHLH repertoire

The above searches and analyses allowed definition of families for the 51 obtained AmbHLHs. This figure is comparable with 52, 50 and 59 bHLH members in the domestic silkworm, red flour beetle and fruit fly, respectively (Table 3). It can be seen that all of these four insects lack genes of families ASCb, NeuroD, Oligo, MyoRb, Figα and Mad, and many of the families have the same number of genes. Major differences were seen in the number of genes of the H/E(spl) family. *D. melanogaster* have 11 to 12 H/E(spl) genes while other insects have 5 to 6. *A. mellifera* has fewer genes in families ASCa, PTFb and Clock than *D. melanogaster*. It is noteworthy that *A. mellifera* has one more gene in families Mist and USF than other insects. Another feature to be noted is that only 2 members of the family ASCa were found in *A. mellifera* and none were found of the family Delilah. Whether *A. mellifera* does have fewer members of these families, or if it was due to incompleteness of the genome sequences remains for further exploitation.

One gene was found to code for Bmal, 2 for ASCa and 3 for E(spl). This is consistent with previous reports (Rubin et al. 2006; Schlatter and Maier 2005). In our survey, 2 genes coding for the Clock family of transcription factors were identified. It is not known if the AmCLK gene cloned by Rubin et al. (2006) is one of them, since no sequence information was available for AmCLK.

#### Expression of AmbHLH genes

A tblastn search with the identified AmbHLH sequences against *A. mellifera* EST databases in GenBank indicated that 10 of them met the searching criterion (Table 4). They are AmOli, AmHand, AmSCL, AmMax, AmUSF2, AmCrp, AmClkl, AmTgo, AmE(spl)2 and AmE(spl)3. Table 4 indicates that the expression of these 10 AmbHLH genes was mainly seen in head tissue. The reason why only 10 AmbHLHs were found to have corresponding EST sequences was probably due to a relatively small deposit of the honey bee EST database which had 78,085 EST sequences as compared to 541,595 for *D. melanogaster* and 4,850,243 for the mouse.

**Table 4.**
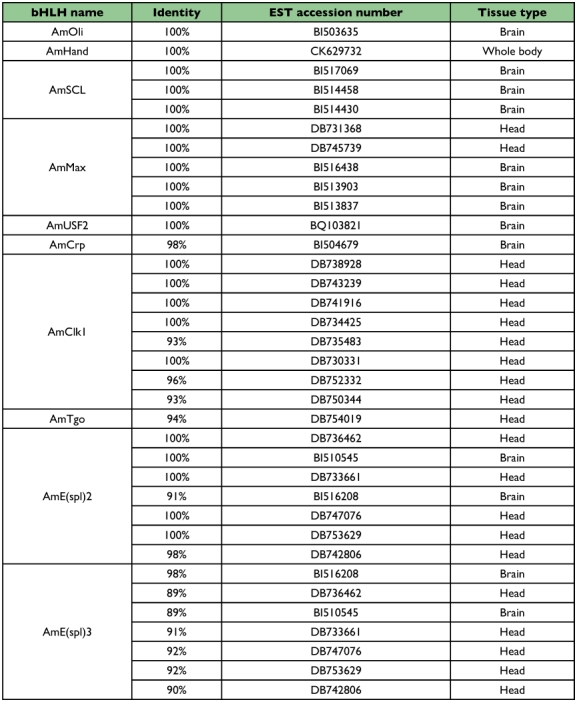
EST sequences of 10 identified AmbHLHs.

### Conclusions

By using the 45 representative bHLH domains and 59 identified DmbHLHs as query sequences, we identified 51 bHLHs from *A. mellifera* genome sequences. It was found necessary to use *D. melanogaster* bHLH sequences as query sequences. This helped us to identify 1 additional bHLHs in *Apis mellifera*. It was also advantageous to use *D. melanogaster* bHLHs of known families to help determine the orthologous genes for *A. mellifera*. Since the 45 representative bHLH domains were mainly from the mouse (Ledent et al. 2002; Simionato et al. 2007), it was reasonable to assign relationships depending on results from *D. melanogaster* when the results from phylogenetic analyses with both representative bHLH domains and DmbHLH motifs did not accord with each other. For instance, in-group analyses with 22 representative bHLH domains suggested orthologous families of PTFa for AmPTFb with bootstrap support of 56 to 73. But ingroup analyses with 24 *D. melanogaster* bHLHs suggested PTFb with much higher bootstrap support (71 to 98) (Table 1). Therefore, PTFb was considered to be the orthologous family for that sequence.

## Abbreviations

ASC -: Achaete-Scute Complex
Ngn -: Neurogenin, H/E(spl) - Hairy/E(spl)
Hs -: Homo sapiens
Mm -: *Mus musculus*
Bf -: *Branchiostoma floridae* (the Florida lancelet)
